# The identification of high-performing antibodies for RNA-binding protein FUS for use in Western Blot, immunoprecipitation, and immunofluorescence

**DOI:** 10.12688/f1000research.133220.2

**Published:** 2023-06-26

**Authors:** Walaa Alshalfie, Maryam Fotouhi, Riham Ayoubi, Zhipeng You, Kathleen Southern, Peter S. McPherson, Carl Laflamme, NeuroSGC/YCharOS/EDDU collaborative group

**Affiliations:** 1 Department of Neurology and Neurosurgery, Structural Genomics Consortium, The Montreal Neurological Institute, McGill University, Montreal, Québec, H3A 2B4, Canada; 2 The Neuro’s Early Drug Discovery Unit (EDDU), Structural Genomics Consortium, McGill University, Montreal, Québec, H3A 2B4, Canada

**Keywords:** Uniprot ID P35637, FUS, RNA-binding protein FUS, antibody characterization, antibody validation, Western Blot, immunoprecipitation, immunofluorescence

## Abstract

RNA-binding protein Fused-in Sarcoma (FUS) plays an essential role in various cellular processes. Mutations in the C-terminal domain region, where the nuclear localization signal (NLS) is located, causes the redistribution of FUS from the nucleus to the cytoplasm. In neurons, neurotoxic aggregates are formed as a result, contributing to neurogenerative diseases. Well-characterized anti-FUS antibodies would enable the reproducibility of FUS research, thereby benefiting the scientific community.

** **In this study, we characterized ten FUS commercial antibodies for Western Blot, immunoprecipitation, and immunofluorescence using a standardized experimental protocol based on comparing read-outs in knockout cell lines and isogenic parental controls.

We identified many high-performing antibodies and encourage readers to use this report as a guide to select the most appropriate antibody for their specific needs.

## Introduction

Fused-in Sarcoma (FUS) encodes a DNA/RNA-binding protein involved in numerous cellular processes including transcriptional regulation, RNA splicing, RNA transport and DNA repair. ^1^ Predominantly localized in the nucleus, FUS can shuttle between the nucleus and cytoplasm. ^2^ The *FUS* transcript is reported to have multiple domains including an N-terminal Gln-Gly-Ser-Tyr -rich region, an RNA-recognition motif, Arg-Gly-Gly repeat regions, a zinc finger motif and a highly conserved C-terminal NLS. ^3^^–^^5^

Variants in the *FUS* gene have been identified as potential causative factors for amyotrophic lateral sclerosis (ALS), frontotemporal dementia (FTD) and frontotemporal lobar degeneration (FTLD). ^6^^–^^9^ FUS related mutations found in familial ALS/FTD patients are clustered in the C-terminal NLS, causing FUS to be mislocalized and accumulate as aggregates in the cytoplasm of neurons, initiating a pathway that contributes to neurodegeneration. ^6^^,^^7^ FUS function is reduced when aggregates form, but it is not yet known whether this initiates the pathogenic process or if the aggregates are pathogenic. ^10^ Mechanistic studies would be greatly facilitated with the availability of high-quality antibodies.

Here, we compared the performance of a range of commercially-available antibodies for RNA-binding protein FUS and validated several antibodies for Western Blot, immunoprecipitation and immunofluorescence, enabling biochemical and cellular assessment of FUS properties and function.

## Results and discussion

Our standard protocol involves comparing readouts from wild-type (WT) and knockout (KO) cells. ^11^^–^^15^ To identify a cell line that expresses adequate levels of FUS protein to provide sufficient signal to noise, we examined public proteomics databases, namely PaxDB ^16^ and DepMap. ^17^ HeLa was identified as a suitable cell line and thus HeLa was modified with CRISPR/Cas9 to knockout the corresponding *FUS* gene ( Table 1).

**Table 1.** Summary of the cell lines used.

Institution	Catalog number	RRID (Cellosaurus)	Cell line	Genotype
ATCC	CCL-2	CVCL_0030	HeLa	WT
Montreal Neurological Institute	-	CVCL_A8VH	HeLa	*FUS* KO

**Figure 1.**
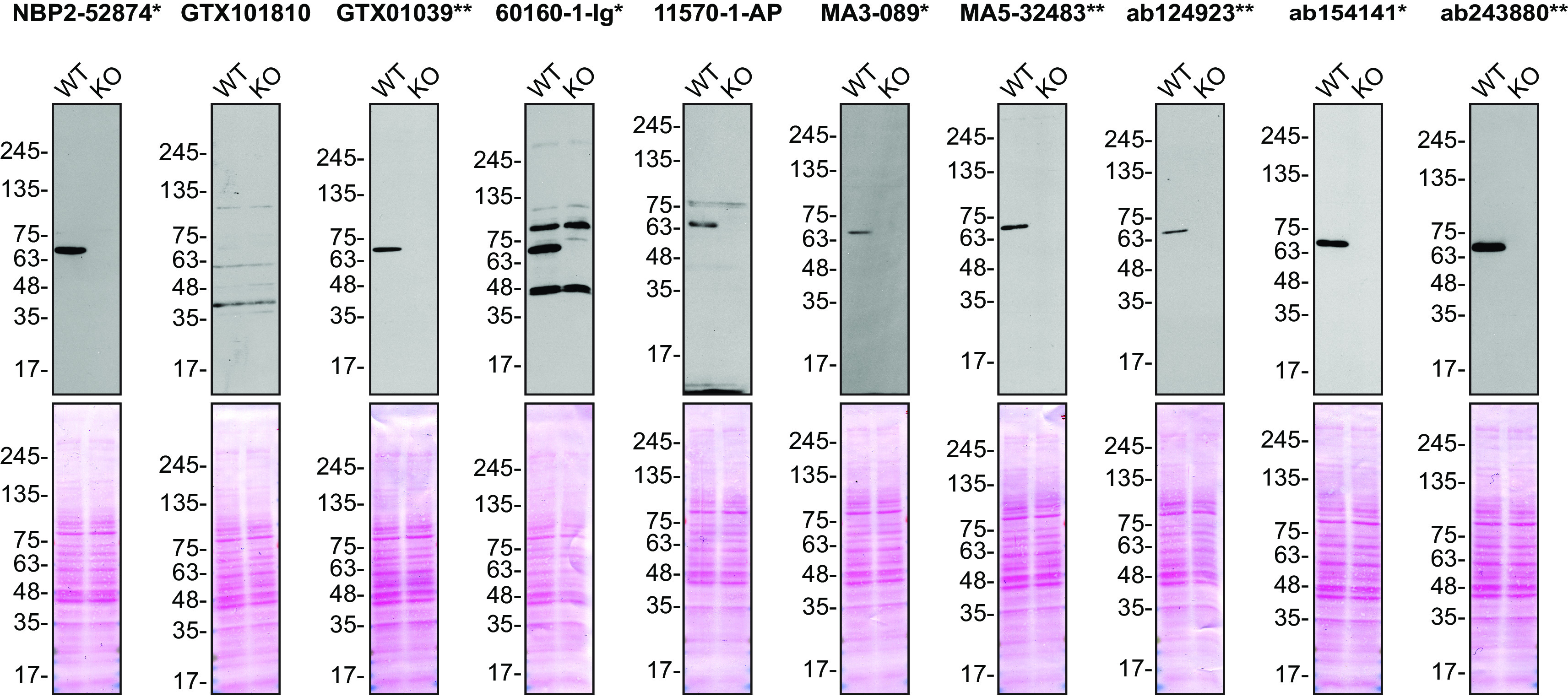
FUS antibody screening by Western Blot. Lysates of HeLa (WT and *FUS* KO) were prepared and 30 μg of protein were processed for Western Blot with the indicated FUS antibodies. The Ponceau stained transfers of each blot are presented to show equal loading of WT and KO lysates and protein transfer efficiency from the acrylamide gels to the nitrocellulose membrane. Antibody dilutions were chosen according to the recommendations of the antibody supplier. An exception was given for antibody GTX101810, which was titrated to 1/3000, as the signal was too weak when following the supplier’s recommendation. Antibody dilution used: NBP2-52874* at 1/1000; GTX101810 at 1/3000; GTX01039* at 1/1000; 60160-1-Ig* at 1/10000; 11570-1-AP at 1/4000; MA3-089* at 1/2000; MA5-32483** at 1/1000, ab124923** at 1/5000; ab154141* at 1/1000; ab243880** at 1/1000. Predicted band size: 53 kDa. Observed specific band size: ~70 kDa. *Monoclonal antibody; **Recombinant antibody.

For Western Blot experiments, we resolved proteins from WT and *FUS* KO cell extracts and probed them side-by-side with all antibodies in parallel ^12^^–^^15^ ( Figure 1).

**Figure 2.**
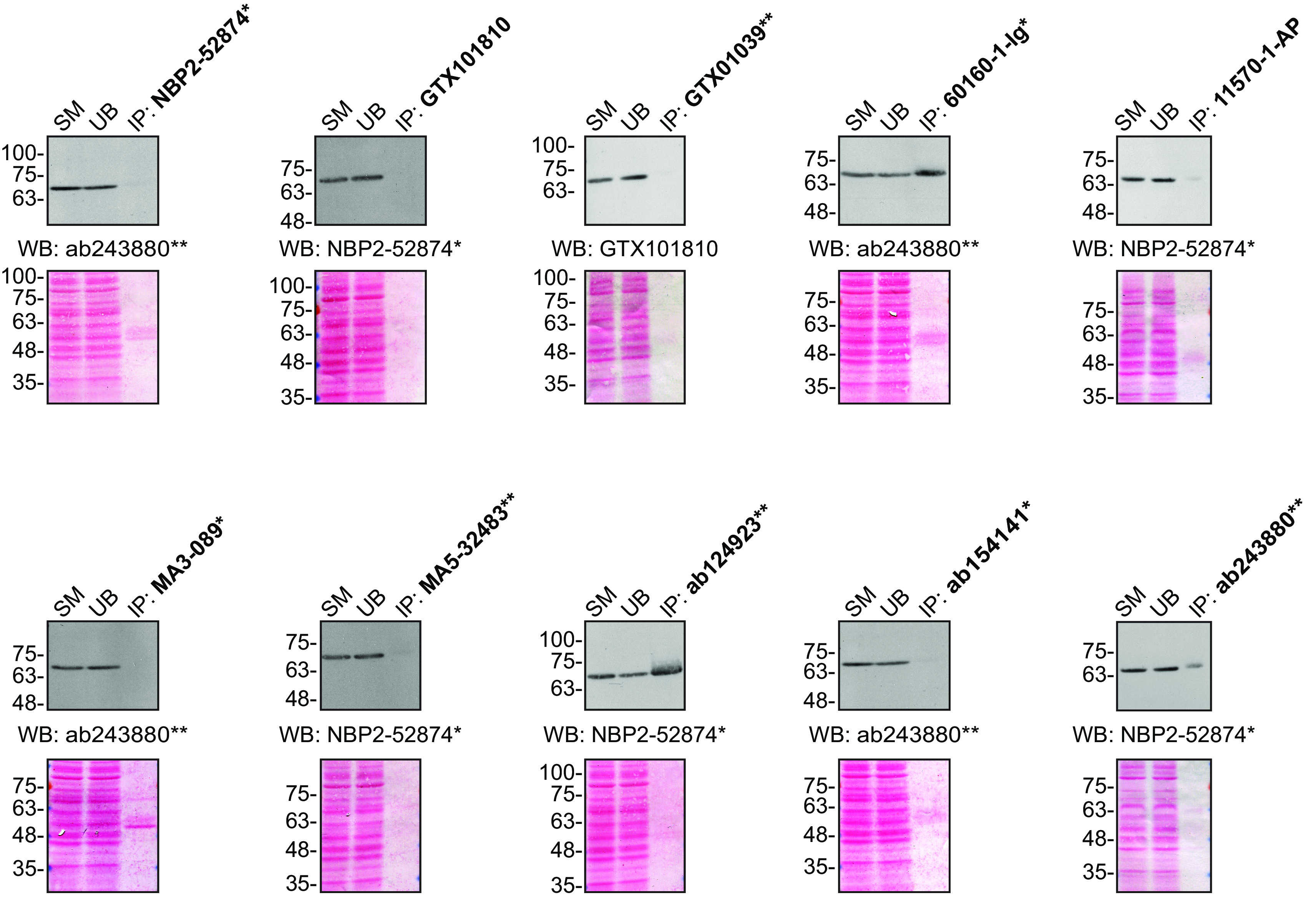
FUS antibody screening by immunoprecipitation. HeLa lysates were prepared, and IP was performed using 1.0 μg of the indicated FUS antibodies pre-coupled to protein G or protein A Sepharose beads. Samples were washed and processed for Western Blot with the indicated FUS antibody. For Western Blot, NBP2-52874* and ab243880** were used at a dilution of 1/2000. The Ponceau stained transfers of each blot are shown for similar reasons as in Figure 1. SM=10% starting material; UB=10% unbound fraction; IP=immunoprecipitated. *Monoclonal antibody; **Recombinant antibody.

For immunoprecipitation experiments, we used the antibodies to immunopurify FUS from HeLa cell extracts. The performance of each antibody was evaluated by detecting the FUS protein in extracts, in the immunodepleted extracts and in the immunoprecipitates ^12^^–^^15^ ( Figure 2).

**Figure 3.**
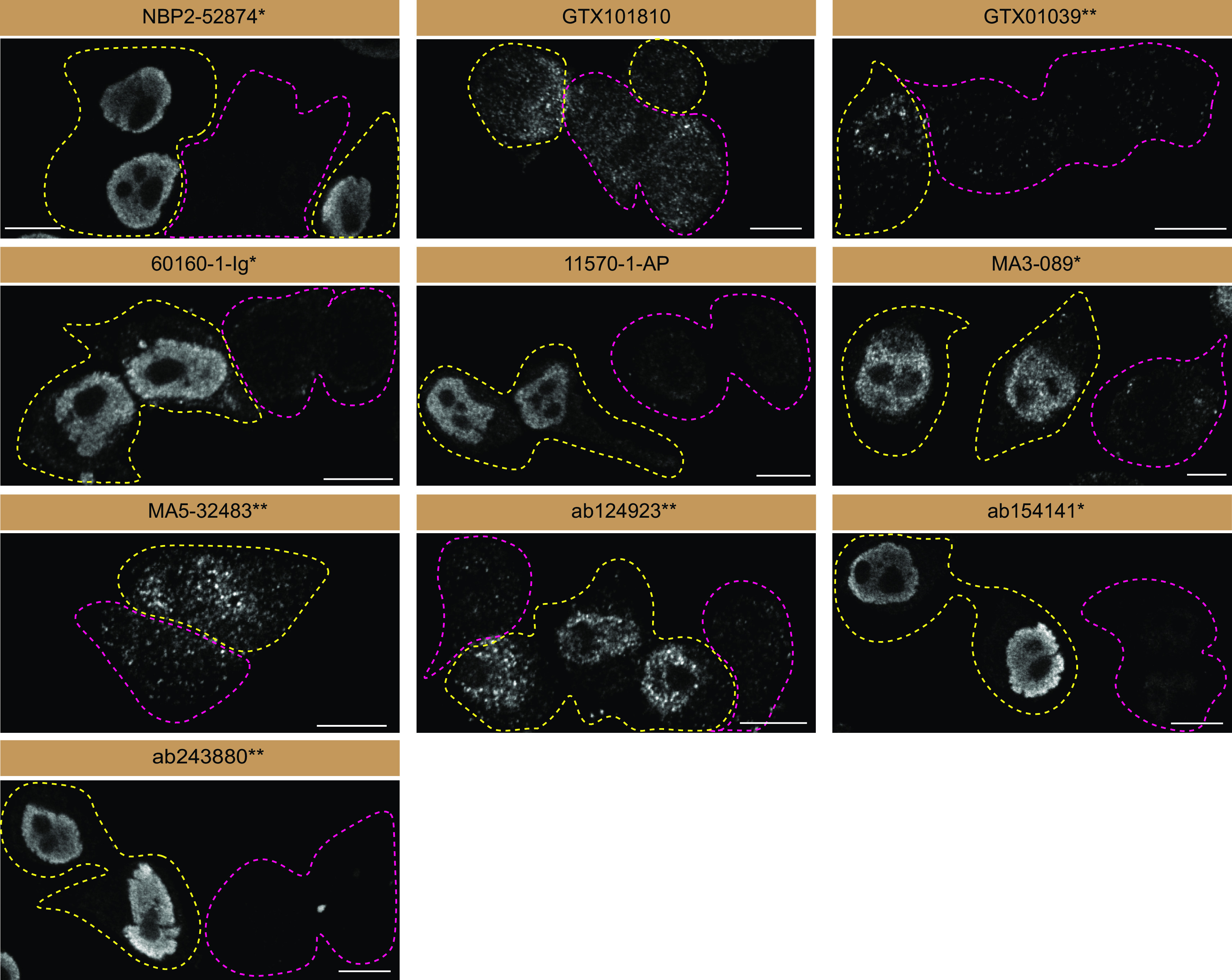
FUS antibody screening by immunofluorescence. HeLa WT and *FUS* KO cells were labelled with a green or a far-red fluorescent dye, respectively. WT and KO cells were mixed and plated to a 1:1 ratio on coverslips. Cells were stained with the indicated FUS antibodies and with the corresponding Alexa-fluor 555 coupled secondary antibody. Acquisition of the green (identification of WT cells), red (antibody staining) and far-red (identification of KO cells) channels was performed. Representative images of the red (grayscale) channels are shown. WT and KO cells are outlined with yellow and magenta dashed line, respectively. Antibody dilutions were chosen according to the recommendations of the antibody supplier. Exceptions were given for antibodies GTX101810, GTX01039*, 60160-1-Ig*, 11570-1-AP, MA5-32483** and ab124923**, which were titrated as the signals were too weak when following the supplier’s recommendations. Antibody dilution used: NBP2-52874* at 1/1000; GTX101810 at 1/700; GTX01039* at 1/1000; 60160-1-Ig* at 1/2000; 11570-1-AP at 1/1000; MA3-089* at 1/1000; MA5-32483** at 1/1000; ab124923** at 1/1000; ab154141* at 1/1000; ab243880** at 1/500. Bars=10 μm. *Monoclonal antibody; **Recombinant antibody.

For immunofluorescence, as described previously, antibodies were screened using a mosaic strategy. ^18^ In brief, we plated WT and KO cells together in the same well and imaged both cell types in the same field of view to reduce staining, imaging and image analysis bias ( Figure 3).

In conclusion, we have screened FUS commercial antibodies by Western Blot, immunoprecipitation and immunofluorescence and identified several high-quality antibodies under our standardized experimental conditions. The underlying data can be found on Zenodo. ^19^^,^^20^

## Methods

### Antibodies

All FUS antibodies are listed in Table 2, together with their corresponding Research Resource Identifiers, or RRID, to ensure the antibodies are cited properly. ^21^ Peroxidase-conjugated goat anti-rabbit and anti-mouse antibodies are from Thermo Fisher Scientific (cat. number 65-6120 and 62-6520). Alexa-555-conjugated goat anti-rabbit and anti-mouse secondary antibodies are from Thermo Fisher Scientific (cat. number A21429 and A21424).

**Table 2.** Summary of the FUS antibodies tested.

Company	Catalog number	Lot number	RRID (Antibody Registry)	Clonality	Clone ID	Host	Concentration (μg/μL)	Vendors recommended applications
Bio Techne	NBP2-52874 *	MAB-03520	AB_2885157	monoclonal	CL0190	mouse	1.00	Wb, IF
GeneTex	GTX101810	40366	AB_2036972	polyclonal	-	rabbit	0.70	Wb
GeneTex	GTX01039 *	822100287	AB_2888934	monoclonal	JJ09-31	rabbit	1.00	Wb, IF
Proteintech	60160-1-Ig *	10017695	AB_10666169	monoclonal	3A10B5	mouse	2.36	Wb, IP, IF
Proteintech	11570-1-AP	00086256	AB_2247082	polyclonal	-	rabbit	0.90	Wb, IP, IF
Thermo Fisher Scientific	MA3-089 *	VB301448	AB_2633334	monoclonal	1FU-1D2	mouse	not provided	Wb, IF
Thermo Fisher Scientific	MA5-32483 **	VL3152611	AB_2809760	recombinant-mono	JJ09-31	rabbit	1.00	Wb, IF
Abcam	ab124923 **	GR85761-9	AB_10972861	recombinant-mono	EPR5812	rabbit	0.15	Wb, IF
Abcam	ab154141 *	GR3368481-1	AB_2885092	monoclonal	CL0190	mouse	1.00	Wb, IF
Abcam	ab243880 **	GR3376392-2	AB_2885123	recombinant-mono	BLR023E	rabbit	not provided	Wb, IP, IF

Wb=Western Blot; IF=immunofluorescence; IP=immunoprecipitation.

* Monoclonal antibody.

** Recombinant antibody.

### CRISPR/Cas9 genome editing

The HeLa *FUS* KO clone was generated with low passage cells using an open-access protocol available on Zenodo.org: https://zenodo.org/record/3875777#.ZA-Rxi-96Rv. Two guide RNAs were used to introduce a STOP codon in the *FUS* gene (sequence guide 1: AGGGAGUCACAAAAGCCACC, sequence guide 2: GGUACGGUGGUGUUGAUGUC).

### Cell culture

Both HeLa WT and *FUS* KO cell lines used are listed in Table 1, together with their corresponding RRID, to ensure the cell lines are cited properly. ^22^ Cells were cultured in DMEM high-glucose (GE Healthcare cat. number SH30081.01) containing 10% fetal bovine serum (Wisent, cat. number 080450), 2 mM L-glutamate (Wisent cat. number 609065), 100 IU penicillin and 100 μg/mL streptomycin (Wisent cat. number 450201).

### Antibody screening by Western Blot

Western Blots were performed as described in our standard operating procedure. ^23^ HeLa WT and *FUS* KO were collected in RIPA buffer (50 mM Tris pH 8.0, 150 mM NaCl, 1.0 mM EDTA, 1% Triton X-100, 0.5% sodium deoxycholate, 0.1% SDS) supplemented with 1× protease inhibitor cocktail mix (MilliporeSigma, cat. number P8340). Lysates were sonicated briefly and incubated for 30 min on ice. Lysates were spun at ~110,000 × g for 15 min at 4°C and equal protein aliquots of the supernatants were analyzed by SDS-PAGE and Western Blot. BLUelf prestained protein ladder from GeneDireX (cat. number PM008-0500) was used.

Western Blots were performed with large 5-16% polyacrylamide gels and transferred on nitrocellulose membranes. Proteins on the blots were visualized with Ponceau S staining (Thermo Fisher Scientific, cat. number BP103-10) which is scanned to show together with individual Western Blot. Blots were blocked with 5% milk for 1 hr, and antibodies were incubated overnight at 4°C with 5% bovine serum albumin (BSA) (Wisent, cat. number 800-095) in TBS with 0.1% Tween 20 (TBST) (Cell Signaling Technology, cat. number 9997). Following three washes with TBST, the peroxidase conjugated secondary antibody was incubated at a dilution of ~0.2 μg/mL in TBST with 5% milk for 1 hr at room temperature followed by three washes with TBST. Membranes were incubated with Pierce ECL (Thermo Fisher Scientific, cat. number 32106) prior to detection with the HyBlot CL autoradiography films (Denville, cat. number 1159T41).

### Antibody screening by immunoprecipitation

Immunoprecipitation was performed as described in our standard operating procedure. ^24^ Antibody-bead conjugates were prepared by adding 1.0 μg of antibody to 500 μL of phosphate-buffered saline (PBS) (Wisent, cat. number 311-010-CL) with 0,01% triton X-100 (Thermo Fisher Scientific, cat. number BP151-500) in a 1.5 mL microcentrifuge tube, together with 30 μL of protein A- (for rabbit antibodies) or protein G- (for mouse antibodies) Sepharose beads. Tubes were rocked overnight at 4°C followed by two washes to remove unbound antibodies.

HeLa WT were collected in HEPES buffer (20 mM HEPES, 100 mM sodium chloride, 1 mM EDTA, 1% Triton X-100, pH 7.4) supplemented with protease inhibitor. Lysates were rocked 30 min at 4°C and spun at 110,000 × g for 15 min at 4°C. One mL aliquots at 1.0 mg/mL of lysate were incubated with an antibody-bead conjugate for ~2 hours at 4°C. The unbound fractions were collected, and beads were subsequently washed three times with 1.0 mL of HEPES lysis buffer and processed for SDS-PAGE and Western Blot on a 5-16% polyacrylamide gels.

### Antibody screening by immunofluorescence

Immunofluorescence was performed as described in our standard operating procedure. ^12^^–^^15^^,^^18^ HeLa WT and *FUS* KO were labelled with a green and a far-red fluorescence dye, respectively. The fluorescent dyes used are from Thermo Fisher Scientific (cat. number C2925 and C34565). WT and KO cells were plated on glass coverslips as a mosaic and incubated for 24 hrs in a cell culture incubator at 37 ^o^C, 5% CO _2_. Cells were fixed in 4% paraformaldehyde (PFA) (Beantown chemical, cat. number 140770-10ml) in PBS for 15 min at room temperature and then washed 3 times with PBS. Cells were permeabilized in PBS with 0,1% Triton X-100 for 10 min at room temperature and blocked with PBS with 5% BSA, 5% goat serum (Gibco, cat. number 16210-064) and 0.01% Triton X-100 for 30 min at room temperature. Cells were incubated with IF buffer (PBS, 5% BSA, 0.01% Triton X-100) containing the primary FUS antibodies overnight at 4°C. Cells were then washed 3 × 10 min with IF buffer and incubated with corresponding Alexa Fluor 555-conjugated secondary antibodies in IF buffer at a dilution of 1.0 μg/mL for 1 hr at room temperature with DAPI. Cells were washed 3 × 10 min with IF buffer and once with PBS. Coverslips were mounted on a microscopic slide using fluorescence mounting media (DAKO).

Imaging was performed using a Zeiss LSM 880 laser scanning confocal microscope equipped with a Plan-Apo 40× oil objective (NA = 1.40). Analysis was done using the Zen navigation software (Zeiss). All cell images represent a single focal plane. Figures were assembled with Adobe Photoshop (version 24.1.2) to adjust contrast then assembled with Adobe Illustrator (version 27.3.1).

## Data Availability

Zenodo: Antibody Characterization Report for RNA-binding protein FUS, https://doi.org/10.5281/zenodo.5259944. ^19^ Zenodo: Dataset for the RNA-binding protein FUS antibody screening study, https://doi.org/10.5281/zenodo.7764130. ^20^ Data are available under the terms of the Creative Commons Attribution 4.0 International license (CC-BY 4.0).
