# Supplementary material for: The identification of high-performing antibodies for RNA-binding protein FUS for use in Western Blot, immunoprecipitation, and immunofluorescence
Source: F1000Res. 2023 Jun 26;12:376. Originally published 2023 Apr 6. [Version 2] doi: 10.12688/f1000research.133220.2 (PMC10293799; doi:10.12688/f1000research.133220.2)
Supplement: Peer review history

# Peer review: Reviewer response for version 2

**DOI:** 10.5256/f1000research.151937.r181569

**Published:** 2023-06-26

**Authors:** Ryota Hikiami

**Affiliations:** 1 Department of Therapeutics for protein misfolding diseases, Shiga University, Hikone, Shiga Prefecture, Japan

Thank you for considering my comments and suggestions on your manuscript. I am pleased to say that the majority of the concerns I raised have been adequately addressed.

 I have only a minor suggestion.

- Introduction (P3, L7 ‘familial ALS/FTD’): Let me write again, as my writing does not seem to be coming across well. If literature reference 9 (Van Langenhove et al., 2010 ^1^) includes “familial” ALS/FTD with a ‘family history’, please disregard it. However, if reference 9 pertains to “sporadic” ALS/FTD without a family history, please include references such as "Frameshift and novel mutations in FUS in familial amyotrophic lateral sclerosis and ALS/dementia." as literature on “familial” ALS/FTD.

Thank you once again for your diligent cooperation throughout this process. I truly appreciate your responsiveness and your commitment to addressing the comments and suggestions.

Are sufficient details of methods and materials provided to allow replication by others?

Yes

Is the rationale for creating the dataset(s) clearly described?

Yes

Are the datasets clearly presented in a useable and accessible format?

Yes

Are the protocols appropriate and is the work technically sound?

Yes

Reviewer Expertise:

Molecular biology, neuroscience

I confirm that I have read this submission and believe that I have an appropriate level of expertise to confirm that it is of an acceptable scientific standard.

## References

[1] : Frameshift and novel mutations in FUS in familial amyotrophic lateral sclerosis and ALS/dementia. *Neurology* .2010;75(9) : 10.1212/WNL.0b013e3181f07e0c 807-14 10.1212/WNL.0b013e3181f07e0c 20668259PMC2938970

# Peer review: Reviewer response for version 1

**DOI:** 10.5256/f1000research.146199.r178227

**Published:** 2023-06-23

**Authors:** Ralph H. Kehlenbach

**Affiliations:** 1 Department of Molecular Biology, Faculty of Medicine, GZMB, Georg-August-Universitat Gottingen, Göttingen, Lower Saxony, Germany

In their manuscript „The identification of high-performing antibodies for RNA-binding protein FUS for use in Western Blot, immunoprecipitation, and immunofluorescence”, Alshalfie and coauthors perform a thorough comparison of commercial antibodies against FUS, a protein that has gained a lot of interest in the field of neurodegenerative diseases and also in general molecular cell biology. Since many antibodies do not perform as good as they are advertised, such a comparison is highly welcome. The approach (also using knockout cell lines as control) is solid and the results are clear and very well documented. I only have one suggestion: in Table 2, the authors should include one or several column(s) (next to “vendors recommended application”) with comments reflecting the results of the study (e.g. ab243880 seems to work well in WB, in IP and in IF).

Are sufficient details of methods and materials provided to allow replication by others?

Yes

Is the rationale for creating the dataset(s) clearly described?

Yes

Are the datasets clearly presented in a useable and accessible format?

Yes

Are the protocols appropriate and is the work technically sound?

Yes

Reviewer Expertise:

NA

I confirm that I have read this submission and believe that I have an appropriate level of expertise to confirm that it is of an acceptable scientific standard.

# Peer review: Reviewer response for version 1

**DOI:** 10.5256/f1000research.146199.r177190

**Published:** 2023-06-15

**Authors:** Ryota Hikiami

**Affiliations:** 1 Department of Therapeutics for protein misfolding diseases, Shiga University, Hikone, Shiga Prefecture, Japan

Dear Laflamme C and co-authors,

 Thank you for allowing me to review your manuscript titled "The identification of high-performing antibodies for RNA binding protein FUS for use in Western Blot, immunoprecipitation, and immunofluorescence." Your study investigates the utility of commercially available antibodies targeting FUS, one of the causative genes of ALS, through WB, IP, and IF techniques. The experimental findings presented by the authors are expected to be valuable and beneficial for FUS researchers. While the overall methodology and results are sound, I propose minor revisions.

 1. Introduction (P3, L6-):

 Unlike ALS, FTD with the *FUS* gene mutation is rare. Does the attached literature include cases of familial FTLD? If not, I recommend adding relevant references or modifying the text accordingly.

 2. Figure 3 (P5):

 The mosaic arrangement of WT and KO cells in the image is clear and visually appealing. However, the image coverage appears to be somewhat limited. I suggest expanding the image coverage or adding additional images to enhance the readers' understanding. 

 I hope these suggestions are helpful in improving the quality and impact of your manuscript. Thank you again for considering my feedback, and I look forward to seeing the revised version.

 Best regards,

 Ryota Hikiami

Are sufficient details of methods and materials provided to allow replication by others?

Yes

Is the rationale for creating the dataset(s) clearly described?

Yes

Are the datasets clearly presented in a useable and accessible format?

Yes

Are the protocols appropriate and is the work technically sound?

Yes

Reviewer Expertise:

Molecular biology, neuroscience

I confirm that I have read this submission and believe that I have an appropriate level of expertise to confirm that it is of an acceptable scientific standard, however I have significant reservations, as outlined above.

# Response

**Published:** 2023-06-21

**Authors:** Kathleen Southern

**Affiliations:** McGill University, Canada

Dear Ryota Hikiami,

 Thank you for your thorough review of this Data Note which analyses the performance of commercial antibodies for RNA binding protein FUS, a potential causative gene in many neurodegenerative diseases.

 To answer your first point of feedback, literature reference 9, which refers to the publication by Van Langenhove et al., performs a mutational analysis of *FUS* in patients with cases of FTLD. To further elucidate this fact, we will be submitting a revised version with modified text to include FTLD in the list of neurodegenerative diseases potentially caused by *FUS* gene variants.  

 As for the second point, we always include a dataset in our reports, which includes all underlying raw data for the experiments performed (reference 20). This increases the transparency and reproducibility of our work. It also allows viewers to have a better understanding of the results and see what wasn’t included in the figures. As for immunofluorescence, the dataset includes czi files of each antibody under the microscope. We hope this provides the additional image coverage you were looking for.

 Thank you again for your suggestions!
